# Synthesis of polycationic nanoparticles for microbial inhibition and killing

**DOI:** 10.7150/ntno.84574

**Published:** 2023-07-24

**Authors:** Swati Saini, Aruna Kukrety, Pratima Ashok Patel, Umesh Kumar, T. Senthilkumar

**Affiliations:** 1 Polymeric Materials Area, Chemical and Material Sciences Division, CSIR-Indian Institute of Petroleum, Dehradun-248005, India.; 2 Academy of Scientific and Innovative Research (AcSIR), Ghaziabad-201002, India.; 3 Biochemical Science Area, Material Resource Efficiency Division, CSIR-Indian Institute of Petroleum, Dehradun-248005, India.

**Keywords:** Polyelectrolytes, nanoparticles, biocidal activity, antimicrobial coatings

## Abstract

Antimicrobial polymers (AMP) appear to be a promising candidate to deal with the current scenario of bacterial resistance against conventional drugs and antibiotics as they mainly depend on disrupting the bacterial membrane. This work investigates the effect of polycations bearing aromatic and aliphatic pendant cationic groups on the antimicrobial performance of AMP. A radical polymerization strategy was adopted to synthesize two different copolymers and convert them into polycations upon post-modification. Polyelectrolytes were converted into nanoparticles by nanoprecipitation and named PE1 and PE2. Polymers were analyzed by NMR, FT-IR, and gel permeation chromatography (GPC). PE1 and PE2 nanoparticles were uniform, spherical particles from FESEM, size, and zeta potential measurements. The antimicrobial properties of polyelectrolytes were determined against pathogenic *Escherichia coli* (*E. coli*), *Bacillus Subtilis (B. Subtilis), Bacillus Amyloliquefaciens (B. Amyloliquefaciens)* and *Citrobecter Freundii (C. Freundii)* bacterias. The biocidal activity determination studies showed that polyelectrolyte PE2 with aromatic pendant units outperformed PE1 with the aliphatic pendant group. This work highlights the remarkable effect of aromatic segmentation, which provides microbial inhibition, and killing is demonstrated as an antibacterial surface coating.

## 1. Introduction

Contamination via microorganisms is generally concerned about numerous human health-associated sectors, such as dental equipment and hospitals, storage and food packaging, water household sanitation, and purification systems 1-3. The existence of harmful microorganisms in such areas causes a variety of infections and diseases. The prompt development of antibiotic resistance further complicates the situation 4-6. However, antimicrobial agents inhibit the growth of microorganisms or kill microbes. Quaternary ammonium salts/compounds (QAC) covalently incorporated into the polymeric chain are responsible for contact-killing actions 7-9. In recent years, polymeric materials, inorganic metal nanoparticles, or some antibiotics can leach out biocidal agents and are responsible for release-based biocidal action. Similar to QAC, quaternary pyridinium compounds and imidazole derivatives with a heterocyclic ring containing nitrogen atom incorporated polymers also exhibit germicidal activity, and they act through a mechanism similar to QACs 10-13.

Antimicrobial drugs are pharmacological substances that can kill microbes and inhibit the growth of microorganisms 14,15. There are tremendous ways through which various infectious diseases can spread, but approximately 50% of microbial infections are because of contaminated surfaces. The coherence of microbial agents with biomaterial is a potential key factor for initiating an infectious disease. The formation of biofilm on the surface is a significant issue associated with infection 16. So there is a requirement to develop a coating that can prevent biofilm formation or even eliminate the already developed biofilm. There is a huge need for developing an antimicrobial coating that can prevent the adhesion of microbes to the surface or kill the microbes after contact with the surface. Recent research focuses on developing new antimicrobial polymer coating possessing dual biocidal action simultaneously by contact killing and biocidal released action. Biocidal surfaces and materials have better biocidal activity and effect for extended periods. An ideal antimicrobial polymer should have the following characteristics a) exhibit long-term activity, b) broad spectrum for pathogenic microorganisms, c) should be stable and non-toxic, d) cost-effective and easy synthesis route, and e) activity can be regenerated when lost 17.

**Scheme 1:**
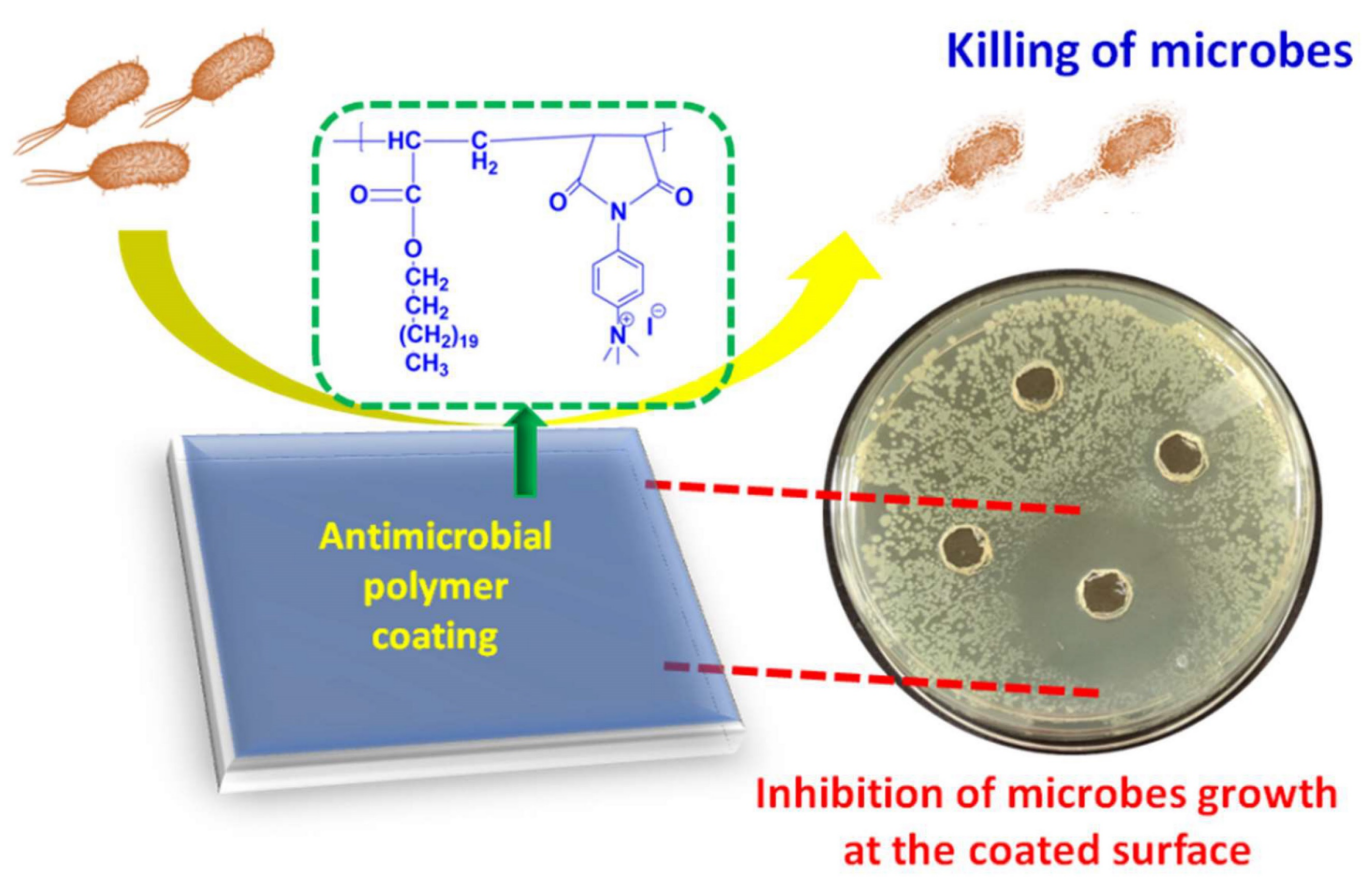
Schematic representation of microbial inhibition by nano polyelectrolyte coatings.

In the present work, two different polyelectrolytes (PE1 and PE2) functionalized with aliphatic and aromatic cations were synthesized by free radical polymerization and post-modification. The synthesized polyelectrolytes were converted to nano polyelectrolytes by the nanoprecipitation method. PE1 and PE2 exhibit excellent water solubility and stability for several days. The antimicrobial performance of PE1 and PE2 were examined and found to inhibit the growth of *E. coli* and *B. Amyloliquefaciens* bacteria. The results inferred that PE2 functionalized with aromatic cations showed excellent antimicrobial inhibition than PE1. These nano polyelectrolytes act as bacteria growth-inhibiting and killing layers over any surface, as shown in **Scheme 1**. Hence these polymers act as bactericidal polymers at a minimum concentration limit of 20 µM.

## 2. Materials and Methods

### Materials

Acrylic acid, toluene, hydroquinone, tetrahydrofuran, and xylene were purchased from SD Fine Chemicals. *p*-Toluene sulfonic acid and maleic anhydride were purchased from SRL. Polyethylene glycol (M_n_=2000) was purchased from TCI chemicals; methyl iodide was purchased from Sigma Aldrich. Ethylene diamine, *p*-phenylene diamine, glutaraldehyde, and 1-docosanol were purchased from Merck and used as received. LB Agar media and Luria Bertani (LB) Broth purchased from Himedia, *E. coli* DH5α, *B. Subtilis, B. Amyloliquefaciens,* and *C. Freundii* strains were obtained from Microbial type culture collection at Chandigarh (MTCC).

### Synthesis of poly(alkylAc-co-MA)

The copolymerization of C_22_ acrylate with maleic anhydride was carried out in toluene by taking the equimolar ratio (1:1) of the two monomers. For this synthesis, 22 mmol (10 g) of C_22_ acrylate and 22 mmol (2.16 g) of maleic anhydride were used as precursors. 0.09 g (0.7 wt%) of benzoyl peroxide was used as a polymerization initiator at 80 °C for 7 h. Toluene was taken as a reaction solvent, and the mixture was refluxed in a 50-mL three-neck round-bottom flask fitted with a condenser. The reaction was allowed to proceed under an inert atmosphere by continuous purging with nitrogen gas. At the end of the reaction, the poly(C_22_Ac-*co*-MA) polymer was precipitated in methanol, filtered out, and washed with methanol several times. The product so obtained was dried in a vacuum oven at 50 °C for 6 h.

### Synthesis of poly(C_22_Ac-co-MA)imide (P1 and P2)

The poly(C_22_Ac-*co*-MA)imide copolymer (P1) was synthesized by reacting equimolar (1:1) quantities of poly(C_22_Ac-*co*-MA) and ethylene diamine. Typically, 10 mmol (5 g) of poly(C*_22_*Ac-*co*-MA) were refluxed for 12 h in xylene in the presence of 10 mmol (0.62 g) ethylene diamine in a round bottom flask fitted with a chilled condenser and a magnetic bar. The resulting poly(C_22_Ac-*co*-MA)imide was precipitated in methanol, filtered, and dried overnight in a vacuum oven at 50°C. Similarly, P2 was also synthesized by a similar procedure with 1.08 g of *p*-phenylene diamine.

### Synthesis of Poly(C_22_-Ac-co-MA) quaternary salt (PE1 and PE2)

The quaternary salt of P1 and P2 was performed by following a reported procedure 18. 300 mg of the weight of P1 and P2 was dissolved in 8 mL of THF, and methyl iodide (excess), was added in four regular intervals till 36 h; the reaction was allowed to continue for 48 h. Finally, the reaction was stopped after 48 h, and the product was dried overnight in a vacuum oven at 50 °C.

### Synthesis of PE1 and PE2 nanoparticles

The polymer nanoparticles were prepared by the nanoprecipitation method 19-21 as follows. 10 mg of P1 was mixed with 20 mg of PEG 2000 and dissolved in THF until they became completely soluble. Mill-Q water was taken in a round bottom flask, and polymer content was added rapidly into water (20 mL) under ultrasonication. THF was removed from the water by bubbling N_2_ gas. The solution is dialyzed against de-ionized water using a dialysis membrane of M_w_ cut-off of 2500. The water was freeze-dried to get a nano polyelectrolyte, PE1. Similar procedures were adopted to synthesize PE2 from P2.

### Characterizations

All the copolymers were extensively characterized by spectroscopic methods. The proton NMR spectra of all the synthesized copolymers were recorded on a Bruker Avance 500 MHz spectrometer (Bruker, United States) by filling the sample in a 5 mm standard NMR tube and using CDCl_3_ as a solvent. Tetramethyl silane (TMS) is used as an internal reference standard. The FT‐IR spectra of the synthesized copolymers were recorded with a PerkinElmer Spectrum-Two FT-IR spectrometer (PerkinElmer, USA) in which a SiC rod is used to generate an infrared source. Spectra were recorded in the mid-IR region, i.e., 4000-400 cm^-1,^ by using the Attenuated Total Reflection (ATR) method. The molecular weight distribution (M_w_/M_n_), number average molecular weight (M_n_), and weight average molecular weight (M_w_) were calculated using a 515 Waters gel permeation chromatography (GPC) system equipped with a 2414 refractive index (RI) detector using Styragel HR 4E column (5 µm, 4.6 x 300 mm) with a molecular weight range of 50 - 100,000 g/mol. HPLC grade THF is used as a mobile phase with a 0.2 mL/min flow rate using narrow-range polystyrene standards. The size and zeta potential measurements of the PE1 and PE2 samples were performed in a Delsa nano C analyzer Beckman Coulter at a concentration of 1 µg/mL. FESEM images were recorded using an FEI, QUANTA 200F scanning electron microscope (Field Electron and Ion Company, USA, a subsidiary of ThermoFisher Scientific) with tungsten filament as an electron source. The polymer was dissolved in HPLC grade THF, dropped on a silicon wafer with a micro syringe, and dried, and the morphology was then recorded. Transmission electron microscopy images were recorded using a JEM-2010 instrument (JEOL, Japan). The accelerating voltage was 200 kV.

### Preparation of bacterial and polyelectrolyte solutions for antibacterial experiments

A single colony of bacteria *(E. coli, B. Substilis, B. amyloliquefaciens,* and *C. freundii*) on a solid LB agar plate was transferred to 10 mL of liquid LB culture medium in the presence of ampicillin (50 μg/mL) and was grown at 37 ^°^C for 6 h. Bacteria were collected by centrifuging (7100 rpm for 1 min) and then were washed with phosphate buffer saline (PBS, 10 mM, pH=7.4) twice. The supernatant liquid was discarded, and the left Ampr *E. coli* were again suspended in PBS and finally diluted to an optical density of 1.0 at 600 nm (O.D.600 = 1.0). 1.0 mM stock solution of PE1 and PE2 were prepared in water. Further, 20 µM, 40 µM, 60 µM, 80 µM, and 100 µM were prepared as working solutions in autoclaved distilled water by mixing.

### SEM analysis

To further study the antimicrobial performance of polyelectrolytes, SEM analysis was used to monitor the process. After the treatment described in antibacterial experiments, *E. coli* was immediately fixed with 0.5% glutaraldehyde in PBS at room temperature for 30 min. The bacteria were centrifuged, the supernatant was removed, and the pellets were suspended in sterile water. 2-3 µL of *E. coli* suspension was dropped in clean silicon wafers, followed by drying in the air. Once the specimens dried up, 0.1% glutaraldehyde was added to fix it and kept as such for 1 h, and then again 0.5% glutaraldehyde was added and left as such for another 2 h. Further, the specimens were washed sterile water twice with and then were dehydrated by adding ethanol in a graded series (70%, 90%, and 100% for 6 min) and dried. Lastly, the specimens were platinum-coated before being kept in the SEM instrument for the experiment.

### Minimum Inhibitory Concentration (MIC)

Two different bacterial cultures were grown individually in LB broth for 14-16 h until they reached in exponential phase (log phase). Autoclaved LB broth of around 4.5 mL was taken in a sterile culture tube, and polymers PE1 and PE2 were added independently in varying concentrations. The final concentration of PE1 and PE2 in LB broth was 20 µM, 40 µM, 60 µM, 80 µM, and 100 µM. Culture tubes containing media with polymer and control were inoculated with 100μL of *E. coli* DH5α or *B. amyloliquefaciens* diluted to 103-fold and incubated at 37 °C &180 rpm.

### Cell viability assay

50 μL cultures from MIC experiments of *E. coli* DH5α were diluted to 103 folds and spread-plated on a sterile LB agar plate, and incubated at 37 °C for 24 h.

### Well diffusion assay

Four different bacterial cultures were grown in LB broth for 14-16 h till their O.D. reached between 0.6-0.8 at 600 nm. 100 μL of 104-fold diluted culture was spread plated on an LB agar plate. Wells were made on an LB agar plate by sterile tips. 100 μL of polymer PE1 and PE2 were coated at the particular circle of the well. Plates were incubated at 37 °C & 180 rpm for 24 h.

## 3. Results and Discussion

**Scheme 2:**
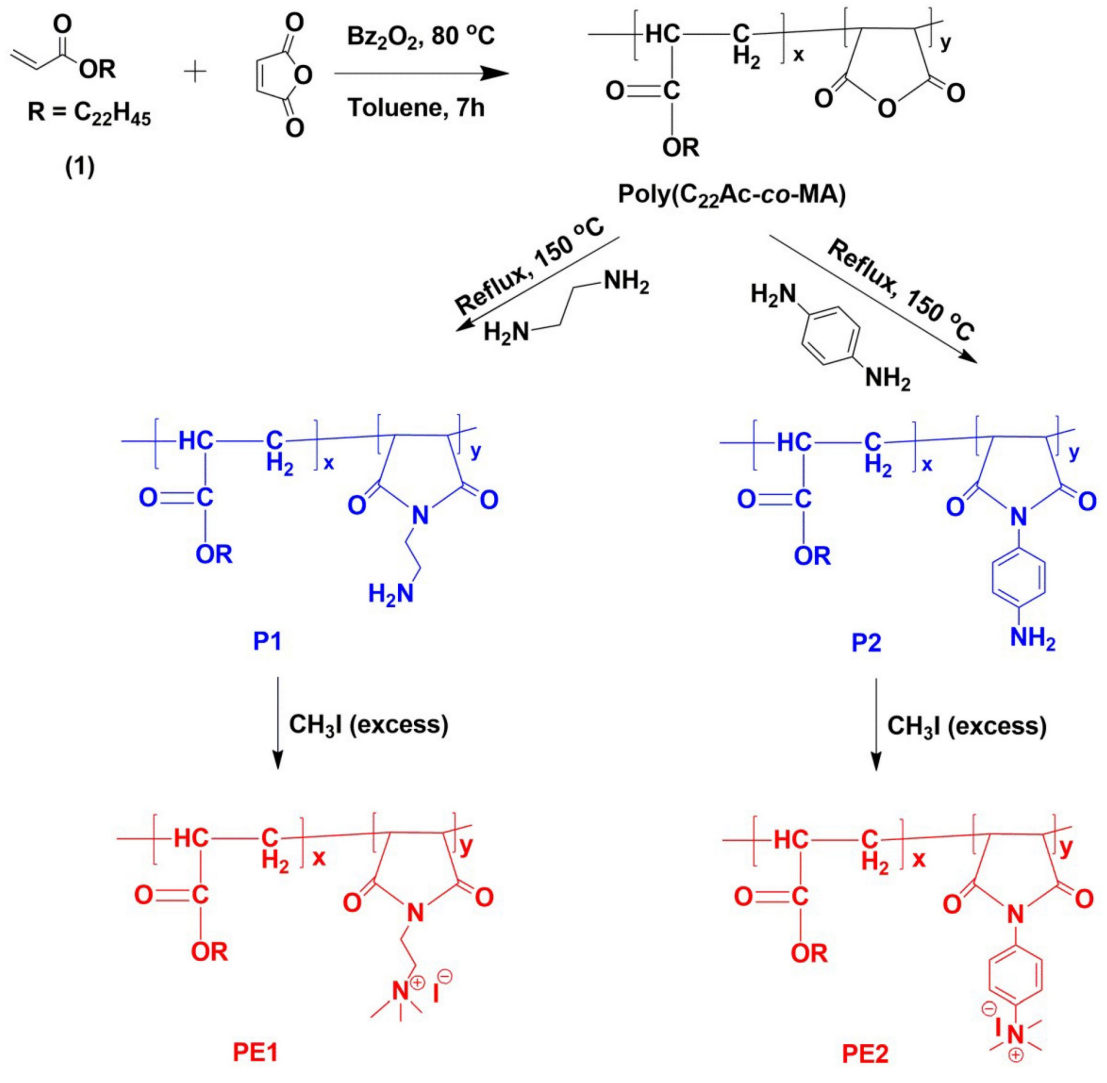
The synthetic route of polyelectrolytes **PE1** and **PE2**.

**Figure 1:**
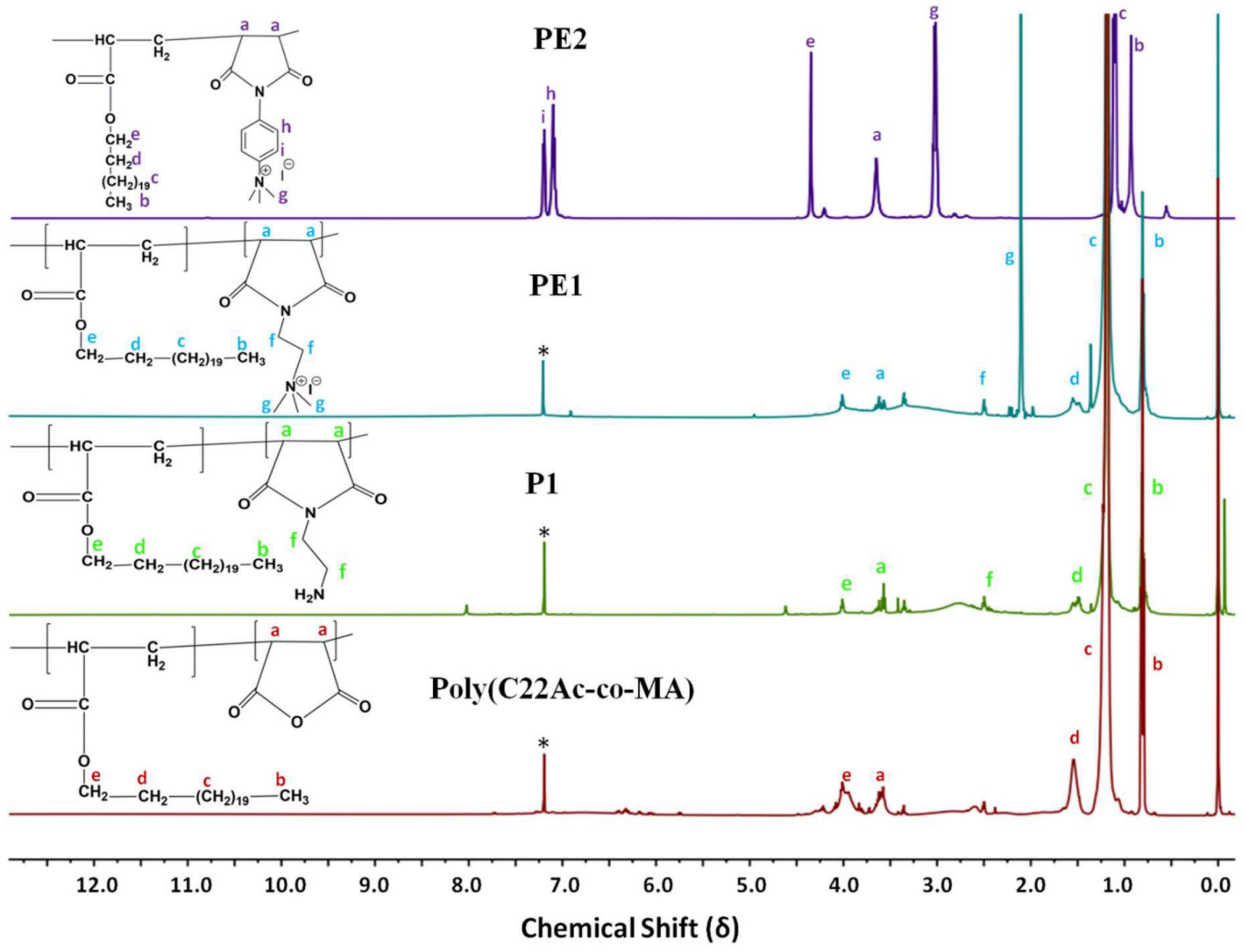
^1^H NMR spectra of Poly(C_22_Ac-*co*-MA), P1, PE1, and PE2 in CDCl_3_.

Polyelectrolytes (PE1 and PE2) were synthesized by a 3-step process, as shown in **Scheme 2**. Firstly, C_22_-alkyl acrylate (1) 22 was copolymerized with maleic anhydride in the presence of Benzoyl peroxide (Bz_2_O_2_) to obtain poly(C_22_-alkyl acrylate-*co*-maleic anhydride) [Poly(C_22_Ac-*co*-MA)]. Poly(C_22_Ac-*co*-MA) was imidized with ethylene diamine and phenylene diamine to produce P1 and P2, respectively. P1 and P2, upon N-alkylation with excess methyl iodide, produce PE1 and PE2, respectively. The detailed experimental procedure for synthesizing small molecules and all the polymers is described in the experimental section. The molecular weight of the polymers P1 and P2 were estimated from GPC using THF as eluent and tabulated in **Table 1**. The number average molecular weight (M_n_) of the P1 and P2 was 8950 and 7030, with a PDI of 1.27 and 1.5, respectively. Synthesis of the small molecules and polymers was confirmed by NMR spectroscopy. **Figure 1** shows the ^1^H NMR spectra of polymers poly(C_22_Ac-*co*-MA), P1, PE1, and PE2. Peaks at chemical shift 0.9, 1.29, and 1.3 ppm were attributed to the -CH_3_, -CH_2_ (long hydrocarbon chain), and -CH_2_ (acrylate backbone) groups. The peak at chemical shift 3.71 ppm corresponds to the -CH_2_- group (polymer backbone) due to maleic anhydride moiety. An absence of an olefinic double bond region at 5.6 ppm gave clear evidence of the successful synthesis of intermediate poly (C_22_Ac-*co*-MA). In the P1 NMR, the proton next to the imide group appeared at 2.48 ppm. In PE1 and PE2, the methyl proton attached to quaternary carbon moiety appears at 2.21 and 3.05 ppm confirming the quaternary salt formation.

**Figure 2:**
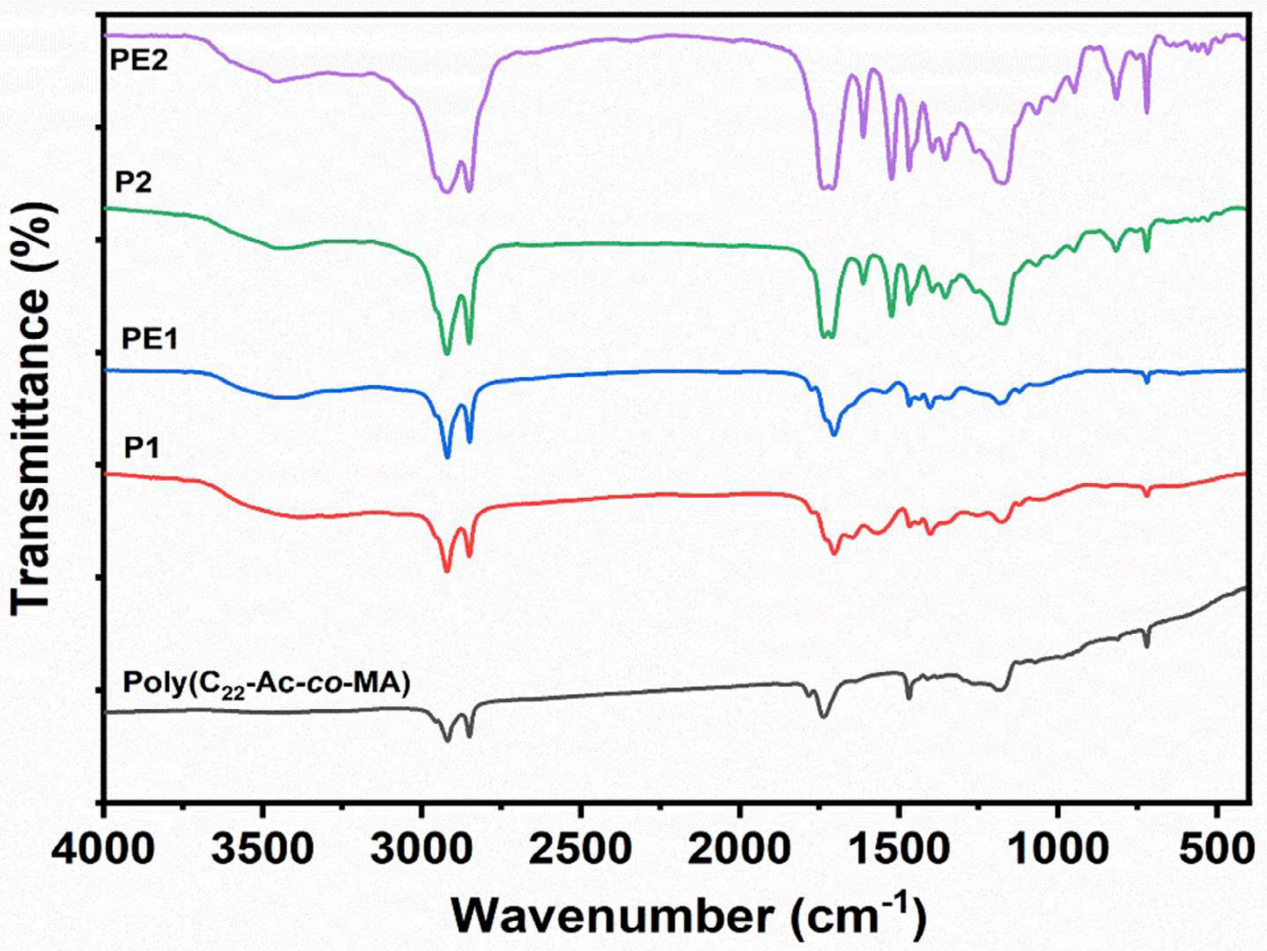
FT-IR spectra of Poly(C*_22_*Ac-*co*-MA), P1, PE1, P2 and PE2.

The FT-IR technique was used for the confirmation of the functional groups in the synthesized polymers poly(C_22_Ac-*co*-MA), P1, PE1, P2, and PE2. The FT-IR spectra of the poly (C_22_Ac-*co*-MA) (**Figure 2**) confirm the successful synthesis of these polymers. In the spectra, strong peaks at 2934 and 2846 cm^-1^ contributed to asymmetric C-H stretching and symmetric C-H stretching and >C=O (ester carbonyl) stretch at 1740 cm^-1^, two new peaks appearing at 1780 and 1857 cm^-1^ corresponding to >C=O stretching vibrations due to anhydride group indicating the successful completion of copolymerization (C_22_Ac with MA). The absence of a C=C stretch peak at 1630 cm^-1^ further confirms the poly (C_22_Ac-*co*-MA) synthesis. Similarly, the FT-IR spectrum of P1 shows C-H asymmetric and symmetric stretch peaks at 2924 and 2854 cm^-1^, respectively, carbonyl >C=O (ester) stretch peak appeared at 1732 cm^-1^, while a weak peak at 1773 cm^-1^ and a strong peak at 1700 cm^-1^ appeared which confirmed the successful imide formation. The peaks at 1780 and 1850 cm^-1^ corresponding to anhydride >C=O stretch completely vanished. The existing peaks at 1466 and 1350 cm^-1^ correspond to C-H bending and C-N stretch, respectively. PE1 and PE2 polyelectrolytes have the same structure with respect to the parent polymer, and the quaternization of amine provides a broader C-H symmetric stretch that appeared at 2956 cm^-1^.

**Figure 3:**
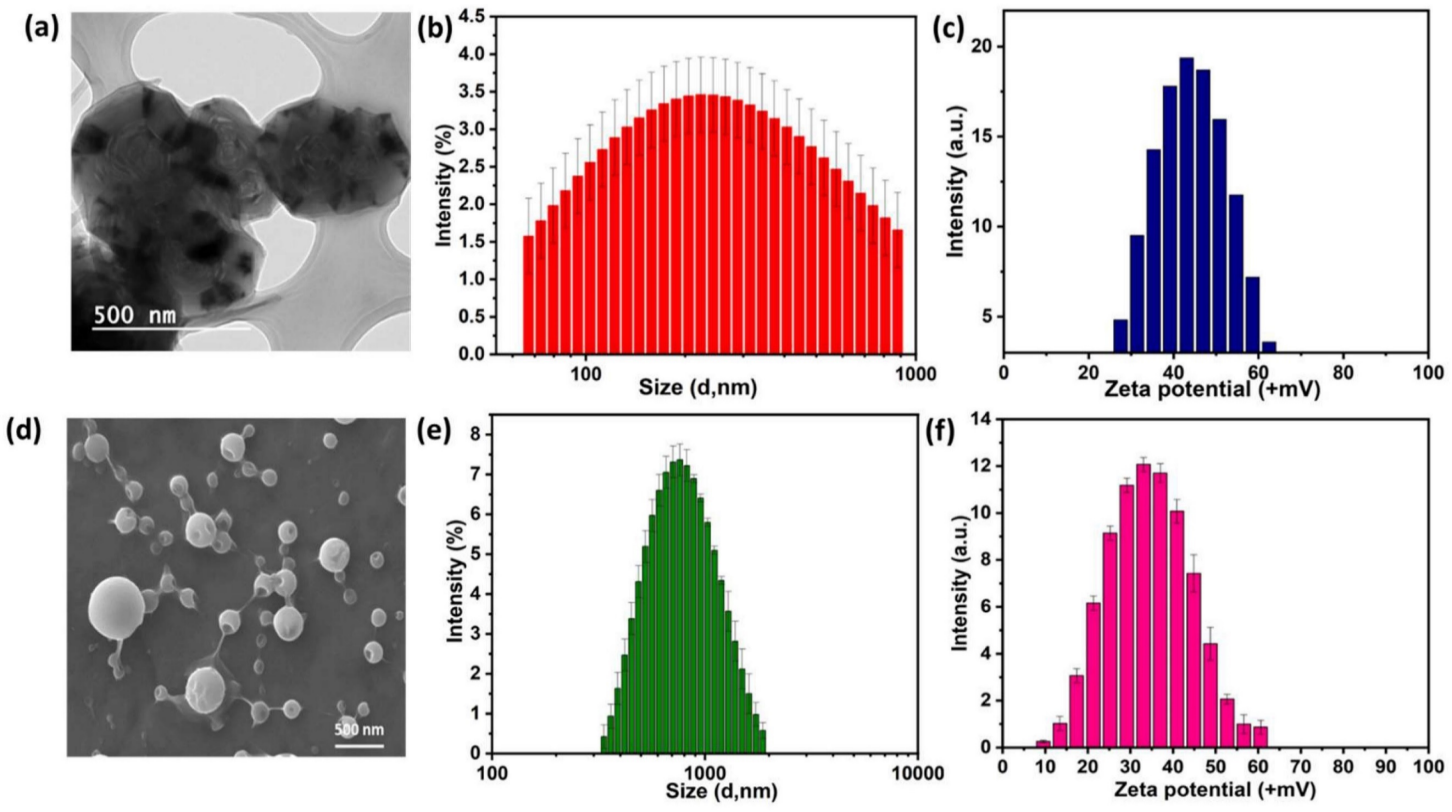
Characterization of PE1 and PE2 nanoparticles: (a) TEM image, (b) particle size and (c) zeta potential measurements of PE1 and (d) FESEM image, (e) particle size and (f) zeta potential measurements of PE2.

The PE1 and PE2 polyelectrolytes were converted into nanoparticles by the nanoprecipitation method 18-20. The Polyelectrolytes (P1 and P2) and PEG2000 were dissolved in THF and poured into water under sonication with constant stirring. The solution was dialyzed against de-ionized water using a dialysis membrane of M_w_ cut-off of 2500. The obtained nanoparticles solution was freeze-dried and stored at 5 °C for further experiments. The structure, size, and charge of the nanoparticles were confirmed from FESEM, particle size measurements, and zeta potential measurements, respectively. Accordingly, Figure 3a-c shows the nanoparticle characterization of the PE1 copolymer. PE1 exhibits a spherical morphology with average particle size and charge of 340 nm and +43.6 mV, respectively. Similarly, Figure 3d-f shows the nanoparticle characterization of the PE2. PE2 also exhibits a spherical morphology with average particle size and charge of 820 nm and +35.4 mV, respectively. Zeta potential is the surface charge over the nanoparticle surface that can greatly influence the particle stability in suspension through the electrostatic repulsion between particles. It can also determine nanoparticle interaction in vivo with the cell membrane of bacteria, which is usually negatively charged. The zeta potential value of +35.4 and +43.6 mV confirms the quaternization of the amine and stable nanoemulsion formation in an aqueous medium.

**Figure 4:**
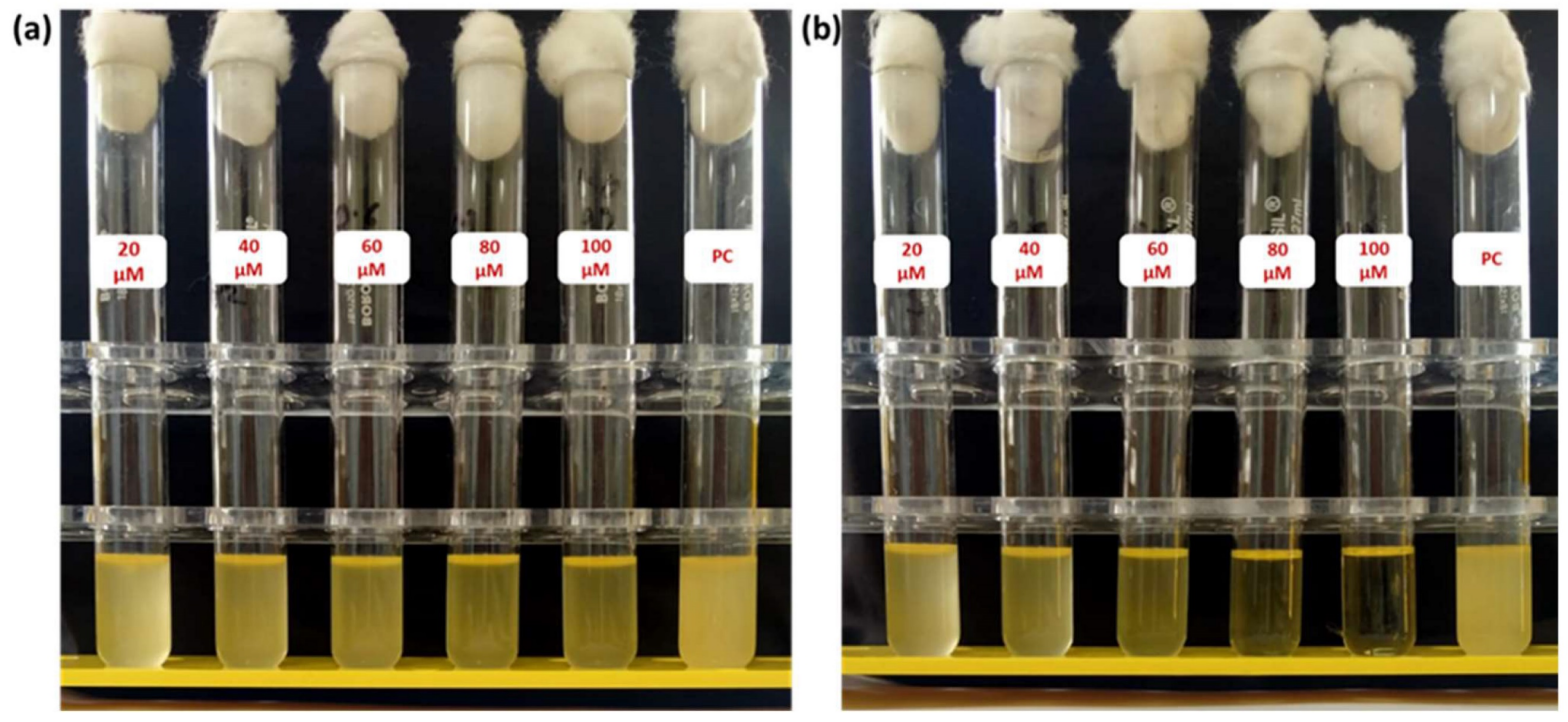
Culture tube experiments of *E. coli* along with PE1 (a) and PE2 (b) along with positive control.

**Figure 5:**
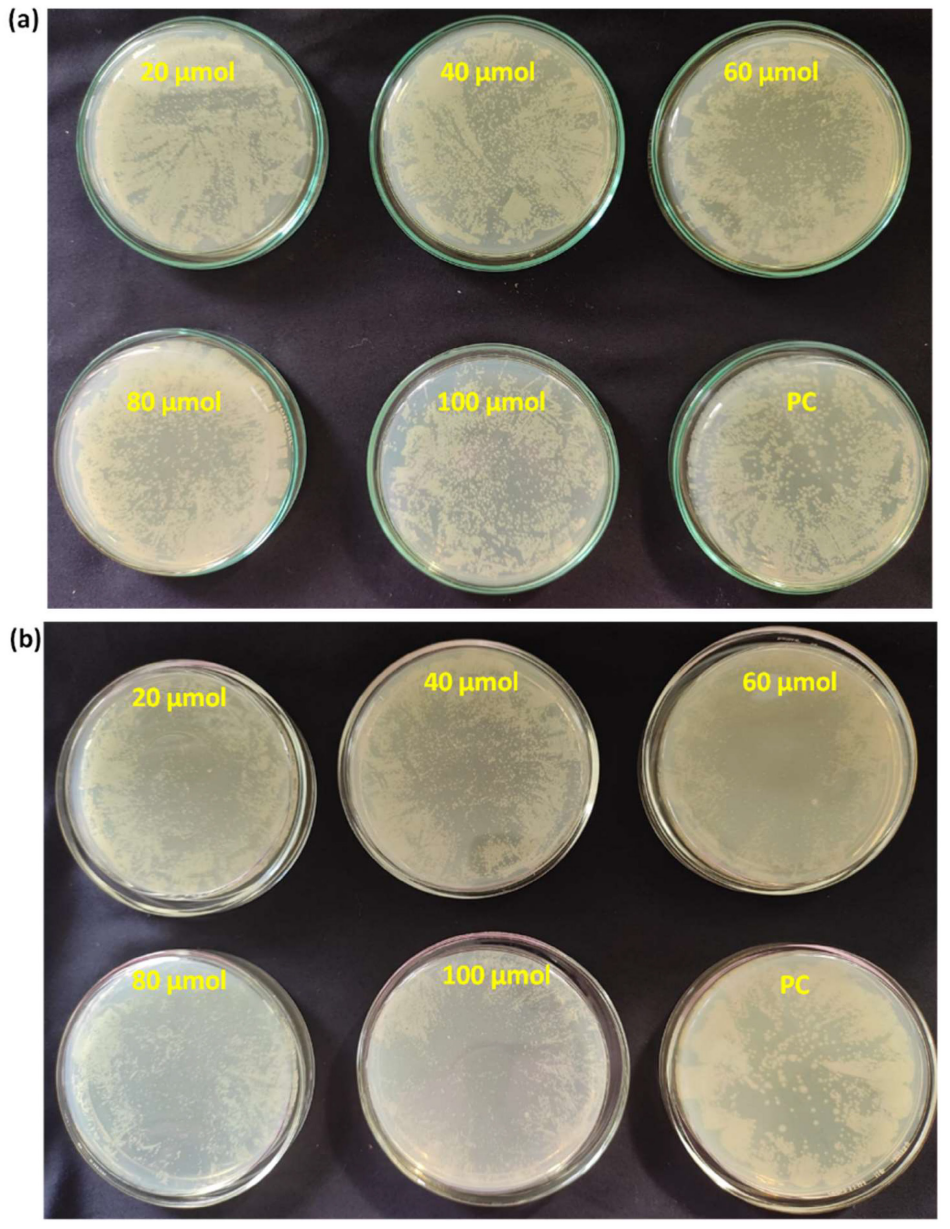
Agar plate experiment with *E. coli DH5α* using PE1 (a) and PE2 (b).

After successfully establishing the structures of the PE1 and PE2 nano polyelectrolytes, the antimicrobial performance of the PE1 and PE2 was studied against four different bacteria such as *E. coli, B. Substilis, B. amyloliquefaciens,* and *C. freundii.* **Figure 4** shows culture tubes containing *E. coli* bacteria along with nano polyelectrolytes PE1 (Figure 4a) and PE2 (Figure 4b). The results showed decreasing pattern of growth (decreasing turbidity) with increasing concentrations of PE1 and PE2. PE2 shows more remarkable *E. coli* inhibition than PE1. It shows its superiority towards microbial growth inhibition. The positive control (PC) indicating PE1 and PE2 has growth-inhibiting effects. PE2 polymer showed a better inhibitory effect compared to PE1 polymer at 60 µM concentration. **Figure 5** shows the microbial growth on all the LB agar plate-containing cultures treated with nano polyelectrolyte PE1 and PE2 for 24 h with varying concentrations (20 µM -100 µM). As the concentration of PE1 and PE2 increased, the size of the colony was much smaller compared to PC indicating polymer PE1, and PE2 are inhibiting the growth of *E. coli DH5α* and are bactericidal as the growth of microbes do not reoccur after the removal of PE1 and PE2. Hence, PE1 and PE2 can act as antimicrobial agents against *E. coli DH5α.* Figure 5(b) shows the remarkable microbial inhibition of nano polyelectrolyte PE2 at a concentration of 60 µM.

**Figure 6:**
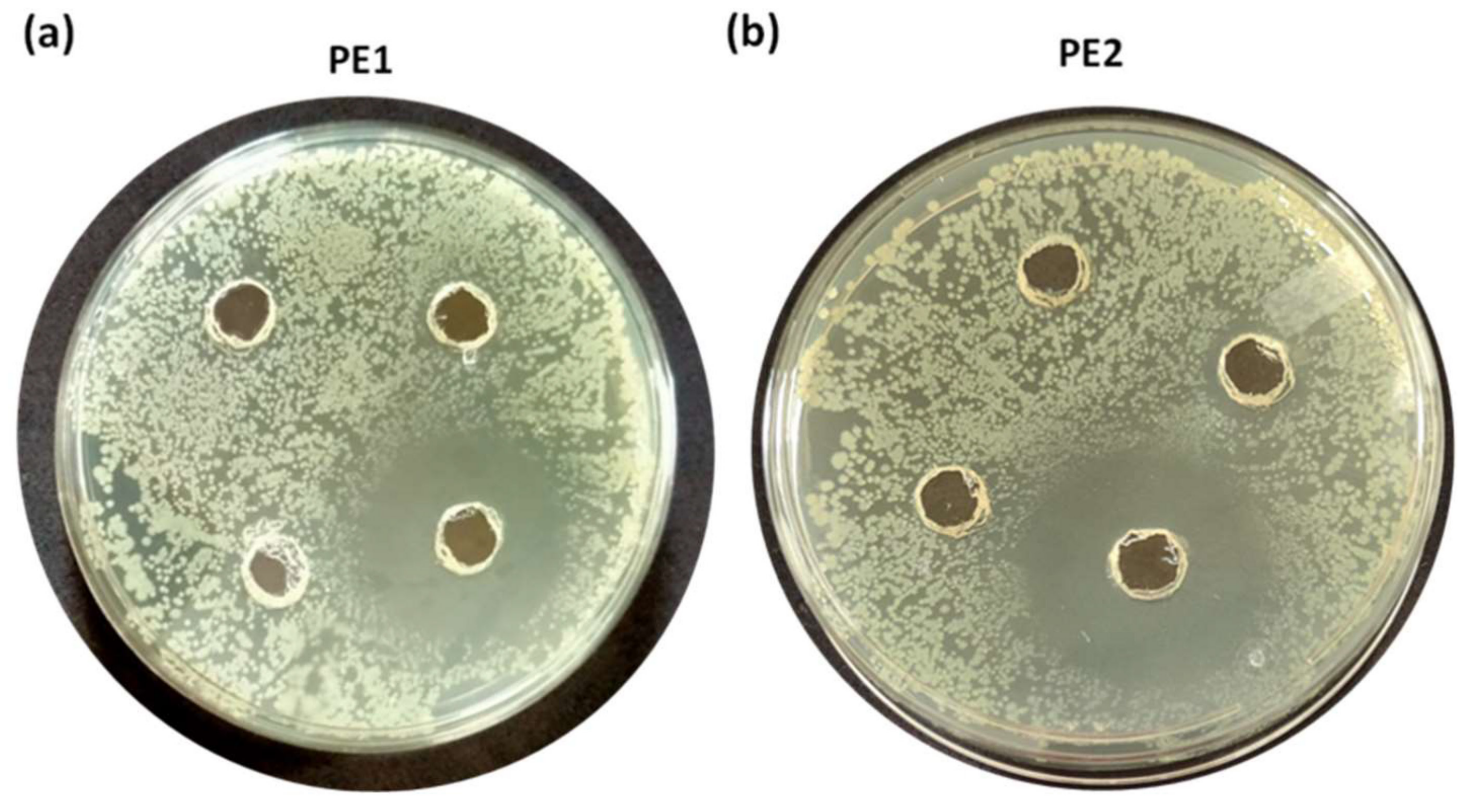
Well, diffusion assay: Selective coating experiments with *E. coli* using PE1(a) and PE2 (b) at a concentration of 100 µM.

**Figure 7:**
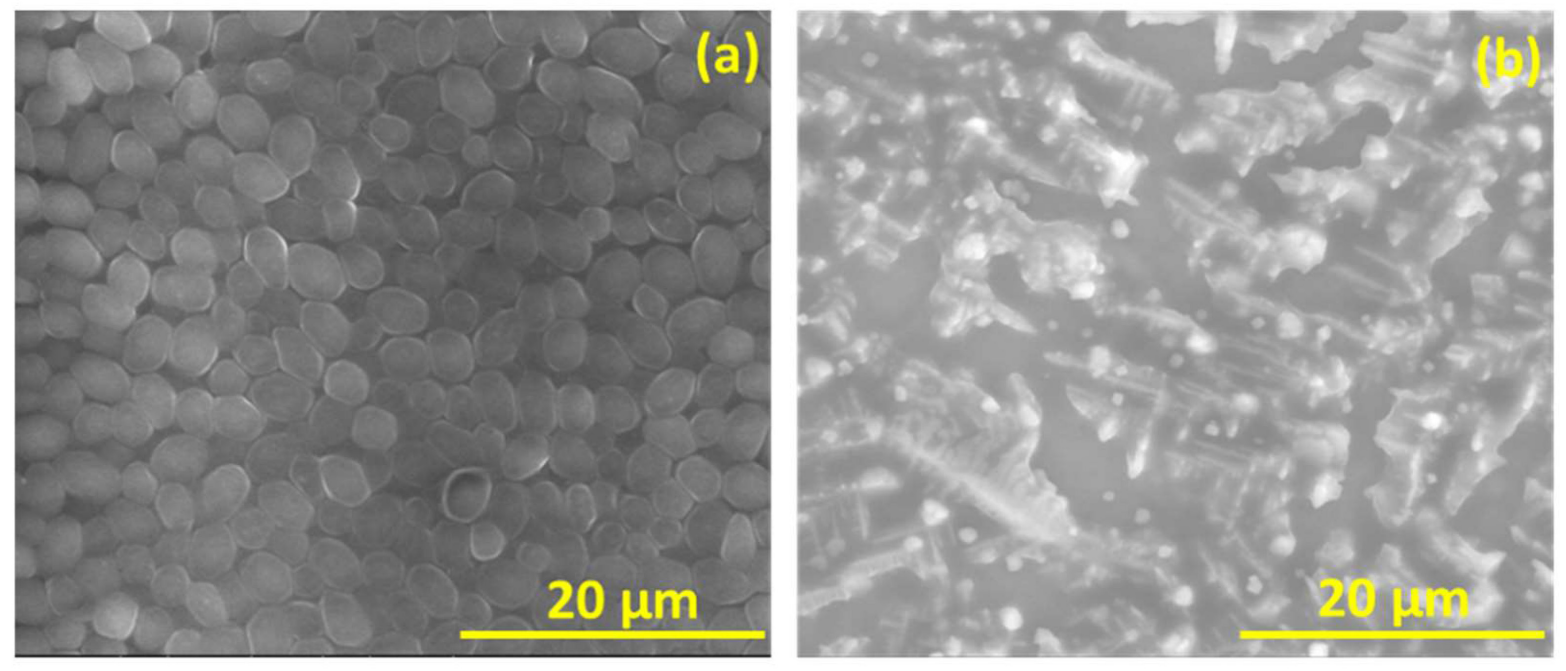
FESEM images of *E.coli* before and after the addition of PE2.

**Figure 8:**
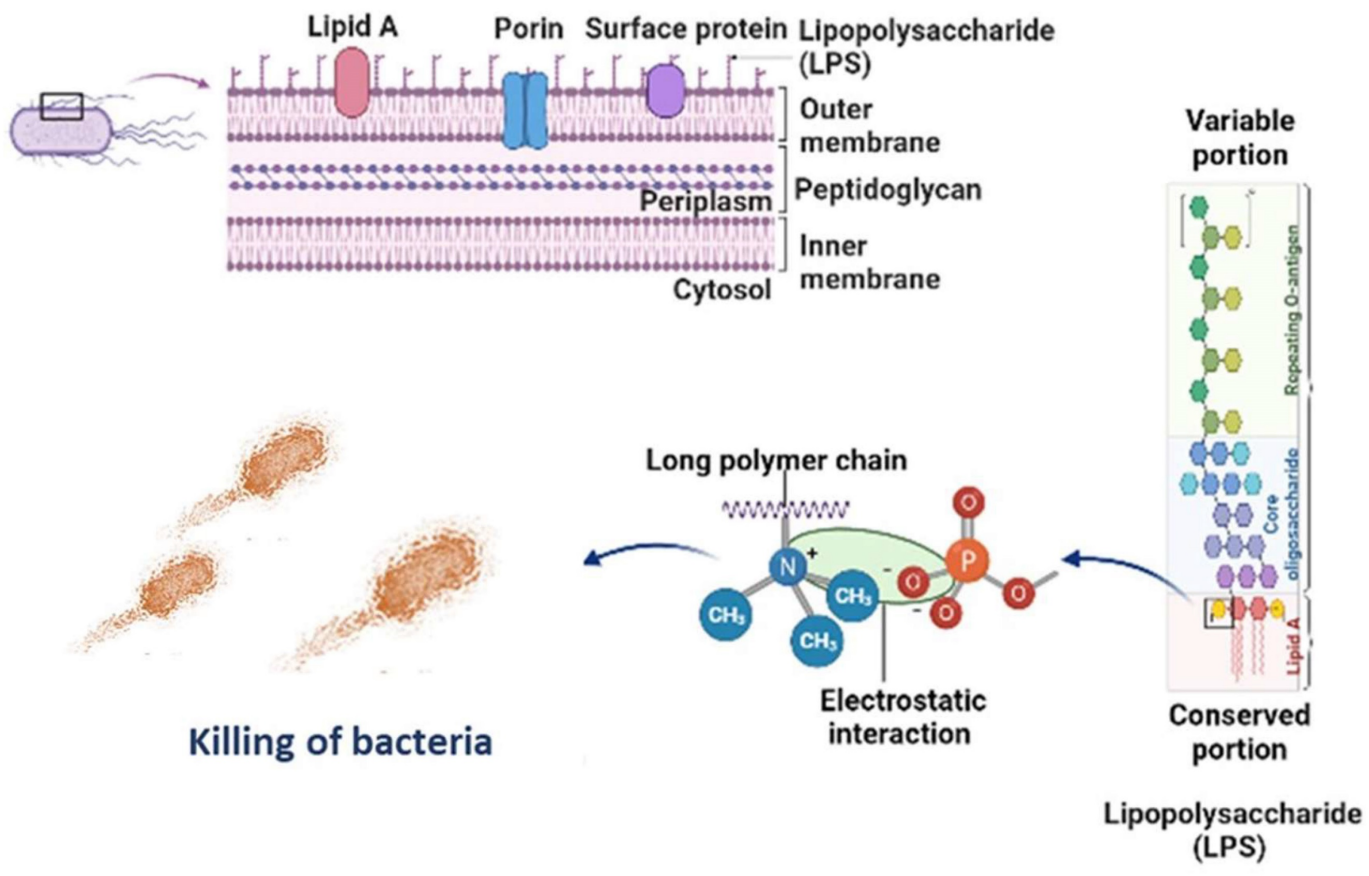
Killing mechanism of *E. coli* with PE2.

**Figure 6** is the well diffusion assay of nano polyelectrolytes PE1 and PE2 at a concentration of 100 µM, which was coated only at the specific circular space of the well. The remaining place was added with conventional agar medium to see the superior performance of these polyelectrolytes as a surface coating agent. The results show no growth of *E. coli* at the specific place where PE1 and PE2 were coated, whereas, at the remaining places, *E. coli* was grown. This indicates the potential of PE1 and PE2 to act as surface sanitization agents at a low concentration of 100 µM. *E. coli* bacterial strains were probed using SEM, and the results are given in **Figure 7**. Figure 7(a) indicates the morphological changes of bacteria without the presence of polyelectrolyte PE2 (control). The control image shows clear well, distinct surface images of the *E. coli* bacteria. The addition of PE2 causes disruption of bacterial cell membranes and release of cytoplasmic content, as shown in Figure 7(b). This demonstrates the potentiality of PE2 as a potential candidate for antimicrobial coatings for microbial inhibition and killing. The plausible killing mechanism of the *E. Coli* bacteria is represented in **Figure 8**. The positively charged polyelectrolyte PE2 is electrostatically attached to the surface of negatively charged *E. Coli* bacteria. The negative charge of *E. Coli* mainly arises from the phosphate ions of the lipopolysaccharide layer. Electrostatic interaction leads to the rupture of the cell membrane, and the entry of polyelectrolytes in the cytoplasm causes severe toxicity to the cell.

**Figure 9:**
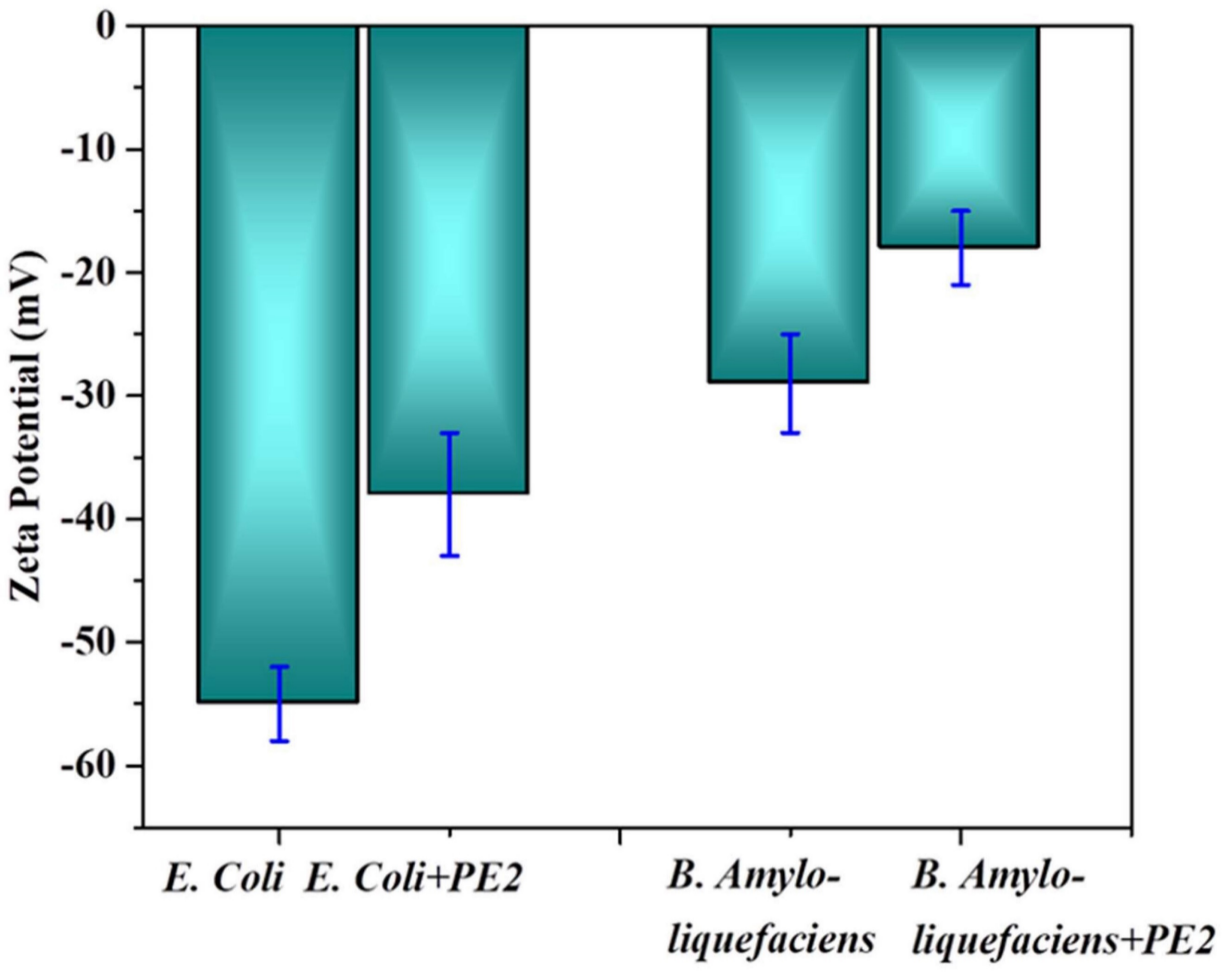
Zeta potential measurement of bacteria with and without PE2.

The electrostatic interaction between *E. Coli* and PE2 is verified with the help of Zeta potential measurements (**Figure 9**). The surface characteristics of *E.Coli* bacteria were probed with zeta potential measurements. The results showed the negative charge of the *E.Coli* bacteria was ς = -55 mV. Upon incubation with PE2 nanoparticle, the negative charge was reduced to ς = -38 mV. Results indicate an electrostatic interaction between the negatively charged *E.Coli* and the polycationic nanoparticle PE2. The surface of *E.coli* is negatively charged because of the dissociation of the phosphate group and carboxyl groups in peptidoglycan and lipopolysaccharides of cell walls. Similarly, *B. Amyloliquefaciens* show a surface charge of -28 mV, and the addition of PE2 causes a reduction of negative surface charge to -17 mV. These results demonstrate the electrostatic interaction between the microbes and the PE2.

**Figure 10:**
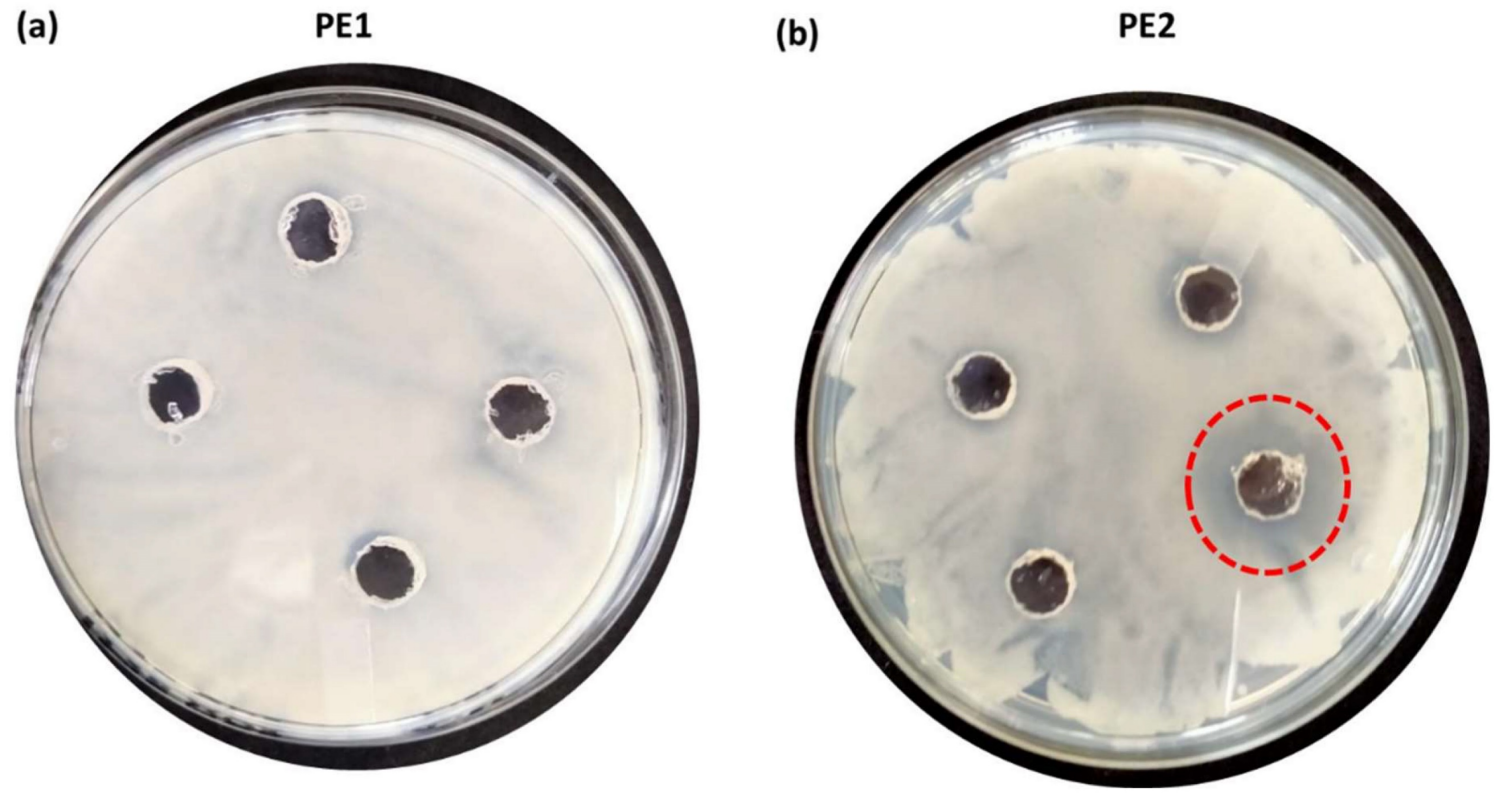
Well, diffusion assay: Selective coating experiments with *B. amyloliquefaciens* using PE1(a) and PE2 (b) at a concentration of 100 µM.

**Figure 11:**
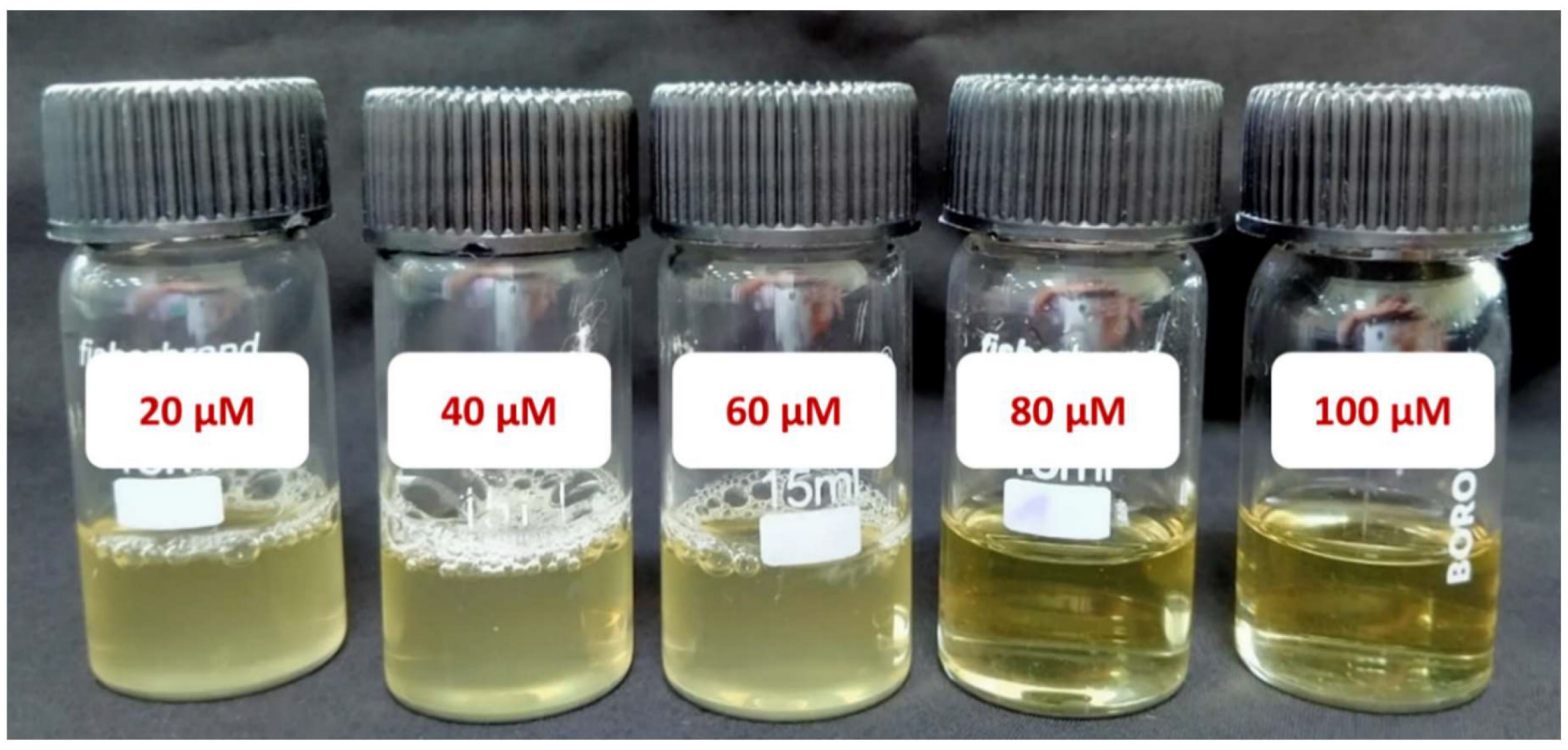
Culture tube experiments of *B. amyloliquefaciens* with PE2.

The antimicrobial applications for other bacteria, including one more Gram-negative *C. Freundii* and two Gram-positive (*B. subtilis* & *B. amyloliquefaciens*) were also carried out by using a well diffusion assay of nano polyelectrolytes. We observed that at 100 µM *B. amyloliquefaciens* is showing growth inhibition in the case of PE2, whereas in the case of PE1 higher concentration (> 1mM) of polymer is required to inhibit the growth of *B. amyloliquefaciens* (**Figure 10**). Culture tube experiments of *B. amyloliquefaciens* with PE2 were also performed and the results showed a decreasing pattern of growth (decreasing turbidity) with increasing concentrations of PE2 (**Figure 11**). The results show up to 60 µM PE2 is not so effective against *B. amyloliquefaciens,* however higher concentration (above 60 µM) is highly effective in killing *B. amyloliquefaciens.*

**Figure 12:**
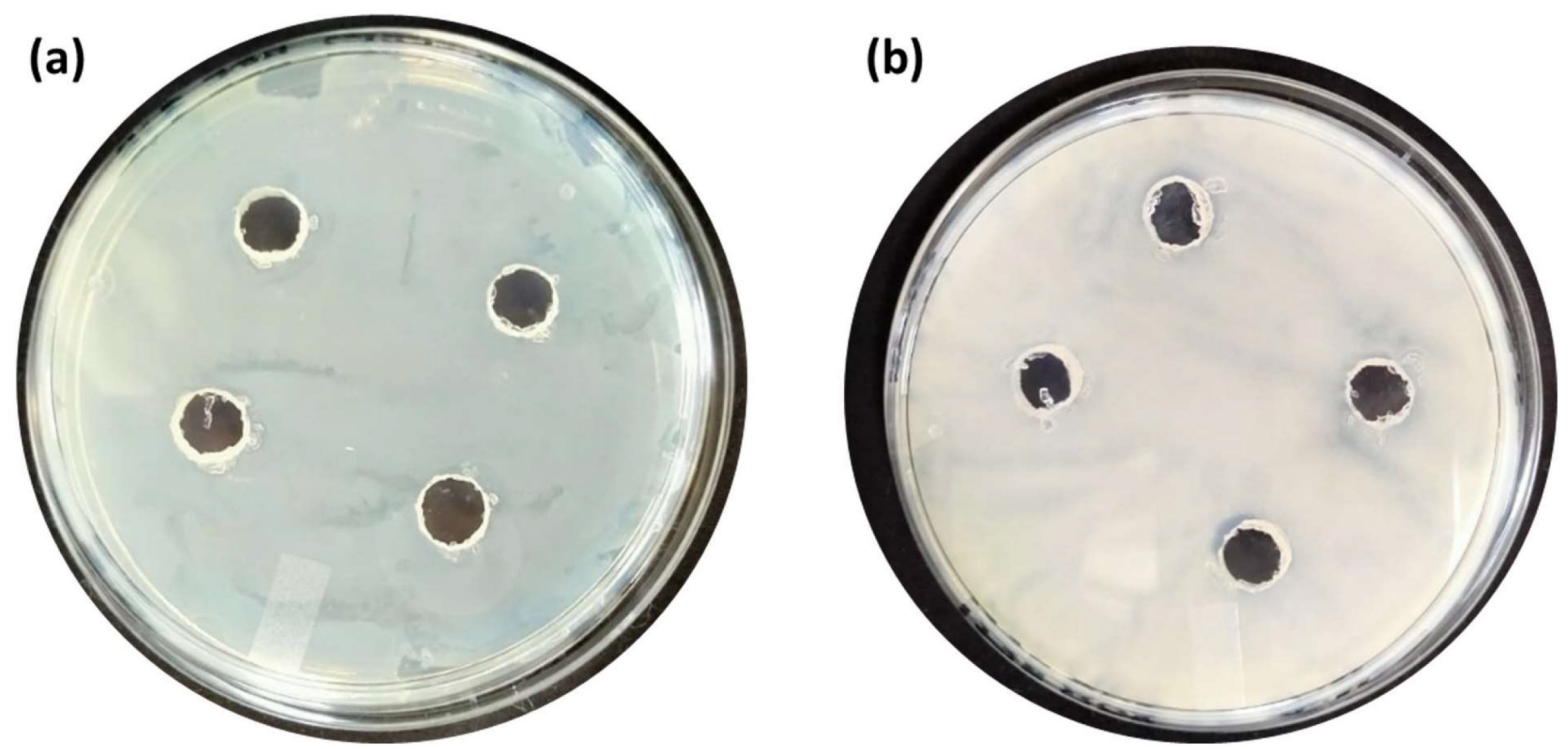
Well, diffusion assay: Selective coating experiments with (a) *B. Subtilis* and (b) *C. Freundii* with PE2 at a concentration of 100 µM.

**Figure 13:**
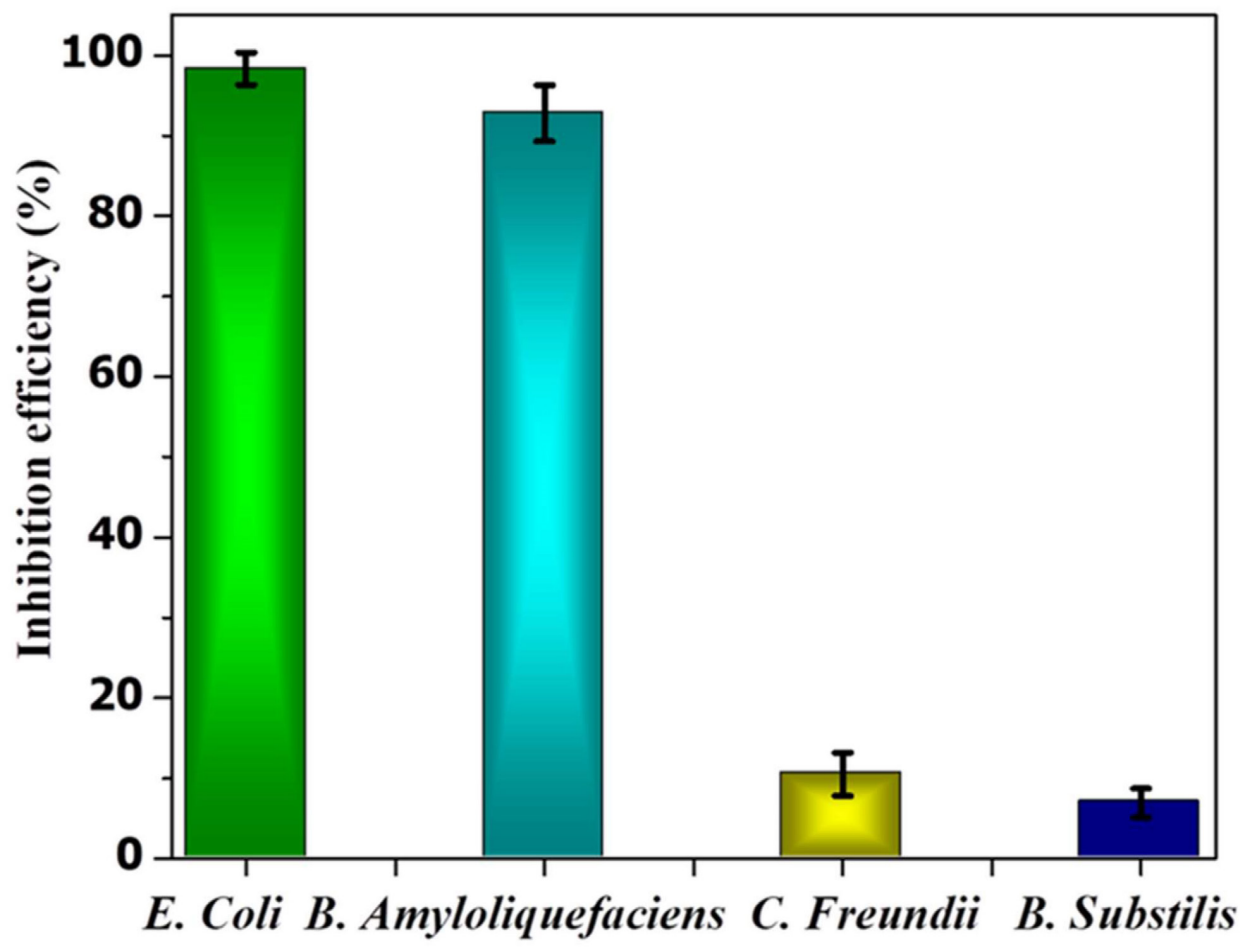
The inhibition efficiency of PE2 at a concentration of 100 µM against various bacterial species.

In the case of *B. subtilis* and* C. Freundii,* even a 100 µM concentration of PE2 is not sufficient to inhibit the growth of bacteria. The zeta potential of the *B. subtilis* and* C. Freundii* are +5 and -2 mV. The addition of PE2 causes a nominal increase in the zeta potential of *B. Subtilis* and* C. Freundii.* This indicates the poor interaction between PE2 and *B. Subtilis* or* C. Freundii* (**Figure 12**). The inhibition efficiency of the PE2 against various bacteria species is plotted in **Figure 13**. The results show that bacteria with negative surface charges such as *E. Coli* and *B. Amyloliquefaciencs* can be effectively killed (>98 %) by the synergistic effect of electrostatic interaction and toxicity of PE2. Therefore PE2 can be effectively used for killing bacteria with a negative surface charge.

## 4. Conclusion

In summary, two different polyelectrolytes with aromatic and aliphatic cations were synthesized and post-modified. The obtained polyelectrolytes are converted into nanoparticles by the nanoprecipitation method to produce PE1 and PE2. The polymers and nanoparticles were well characterized by NMR, FT-IR, GPC, FESEM, DLS, and zeta potential measurements. The nanoparticles were tested for antimicrobial performance in the culture tubes and cell culture wells. The results were found to be remarkable bacterial growth inhibition at a concentration of 20 µM. Also, PE2 outperforms in antimicrobial growth resistance compared to PE1 because of its aromatic cationic nature. These polyelectrolytes are demonstrated as surface sanitization agents and antimicrobial coatings at lower concentration ranges.

## Figures and Tables

**Table 1:** The molecular weight details of P1 and P2 from GPC in THF solvent.

Copolymer	M_n_ (g.mol^-1^)	M_w_ (g.mol^-1^)	PDI	Yield (%)
**P1**	8950	11380	1.27	75
**P2**	7030	10656	1.5	72
